# The Work of Desirabode

**Published:** 1847-10

**Authors:** 


*The Work of Desirabode.*—We shall complete the publication of the translation of this elaborate French treatise upon the science and art of dental surgery, in the Library part of our next number. The translation of this work has prevented the publication of several numbers of the Journal at the periods when they should have been issued. The translation being now nearly completed, we shall be able, for the future, to get the numbers out in better time.—*Balt. Ed.*

